# The First Annual Report

**Published:** 1918-02-23

**Authors:** Tom B. Harrison

**Affiliations:** Secretary. Royal West Sussex Hospital, Chichester.


*To the Editor of* The Hospital.

### The First Annual Report.

*To the Editor of* The Hospital.

Sir,—With reference to your remarks *re* balance sheet for 1917 in your last issue, you will no doubt be interested to hear that the accounts referred to have now been duly audited and found correct. I hope I have won the "race." I shall probably be able to send you a copy of audited accounts this week.

Yours faithfully,
Tom B. Harrison, Secretary.
Royal West Sussex Hospital, Chichester.
February 19, 1918.

[See note under this head on p. 433.—Ed. The Hospital.]

